# An immunocompetent patient with a nonsense mutation in *NHEJ1* gene

**DOI:** 10.1186/s12881-019-0784-0

**Published:** 2019-03-21

**Authors:** Hossein Esmaeilzadeh, Mohammad Reza Bordbar, Zahra Hojaji, Parham Habibzadeh, Dorna Afshinfar, Mohammad Miryounesi, Majid Fardaei, Mohammad Ali Faghihi

**Affiliations:** 1 Allergy Research Center, Shiraz University of Medical Sciences, Shiraz, Iran; 2 Department of Allergy and Clinical Immunology, Namazi Hospital, Shiraz University of Medical Sciences, Shiraz, Iran; 3 Hematology Research Center, Shiraz University of Medical Sciences, Shiraz, Iran; 4 Persian BayanGene Research and Training Center, Shiraz, Iran; 5 Student Research Committee, Shiraz University of Medical Sciences, Shiraz, Iran; 6 Genomic Research Center, Shahid Beheshti University of Medical Sciences, Tehran, Iran; 7 Comprehensive Medical Genetic Center, Shiraz University of Medical Sciences, Shiraz, Iran; 8 Department of Medical Genetics, Shiraz University of Medical Sciences, Shiraz, Iran; 9 Center for Therapeutic Innovation, Department of Psychiatry and Behavioral Sciences, University of Miami Miller School of Medicine, Miami, USA

**Keywords:** Severe combined immunodeficiency, Nonhomologous end-joining factor 1, human, Autoimmune hemolytic Anemia, Genetic disorders, Immunologic deficiency syndromes

## Abstract

**Background:**

DNA double-strand breaks (DSBs) are among the most deleterious types of DNA damage. DSBs are repaired by homologous recombination or non-homologous end-joining (NHEJ). NHEJ, which is central to the process of V(D)J recombination is the principle pathway for DSB repair in higher eukaryotes. Mutations in *NHEJ1* gene have been associated with severe combined immunodeficiency.

**Case presentation:**

The patient was a 3.5-year-old girl, a product of consanguineous first-degree cousin marriage, who was homozygous for a nonsense mutation in *NHEJ1* gene. She had initially presented with failure to thrive, proportional microcephaly as well as autoimmune hemolytic anemia (AIHA), which responded well to treatment with prednisolone. However, the patient was immunocompetent despite having this pathogenic mutation.

**Conclusions:**

Herein, we report on a patient who was clinically immunocompetent despite having a pathogenic mutation in *NHEJ1* gene. Our findings provided evidence for the importance of other end-joining auxiliary pathways that would function in maintaining genetic stability. Clinicians should therefore be aware that pathogenic mutations in NHEJ pathway are not necessarily associated with clinical immunodeficiency.

## Background

DNA double-strand breaks (DSBs), resulting in loss of considerable chromosomal regions, are among the most deleterious types of DNA damage. With an estimated rate of ten per day, DSBs can either be caused by DNA damaging agents such as reactive oxygen species or could be a part of physiological DNA recombination taking place in the immune system [1, 2]. V(D)J recombination is a process during which the highly diverse lymphocyte antigen receptors breed. One of the consequences of this process is DSB [3]. If left unrepaired, DSB will induce either apoptosis or cellular dysfunction [4].

DSBs are repaired by homologous recombination or non-homologous end-joining (NHEJ) [5]. Error-prone NHEJ, which is central to the process of V(D)J recombination, is the main pathway of DSB repair system in higher eukaryotes [5, 6]. Considering its major role in the immune system development, deficiency of *NHEJ1* gene products manifests with absence of mature T and B lymphocytes, also known as “severe combined immunodeficiency” (SCID) [7]. Mutations in the *NHEJ1* have been associated with the clinical phenotype of severe combined immunodeficiency with microcephaly, growth retardation, and sensitivity to ionizing radiation (Phenotype MIM # 611291). The patients reported so far have always presented with clinical manifestations such as failure to thrive (FTT), severe growth retardation, microcephaly, and autoimmune hemolytic anemia (AIHA) [8–10]. Herein, we report on a patient who was clinically immunocompetent despite having a pathogenic mutation in *NHEJ1* gene.

## Case presentation

The patient was a 3.5-year-old girl, a product of consanguineous first-degree cousin marriage, who was born at the gestational age of 38 weeks after a normal and uncomplicated pregnancy. She was in good health after delivery with a good APGAR score. Her weight, length and head circumference were 2500 g, 45 cm and 33 cm, respectively. Weight and length were below the 3rd percentile, whereas head circumference was slightly above the 15th percentile according to the national child growth curve. Failure to thrive and proportional microcephaly continued until one year of age but development was good. She presented with jaundice at the age of one year. Laboratory tests showed decreased WBC count (3000/mm^3^, reference range for age: 5000–15,500/mm^3^) with 64% neutrophil and decreased hemoglobin levels (11 g/dL, reference range for age: 12–14 g/dL). Furthermore, lab results revealed an MCV of 88.7 fL, platelet count of 261,000, ESR of 2 mm/h, reticulocyte count of 5.1%, a positive direct Coomb’s test, negative indirect Coomb’s test. Moreover, ACLA, ANA, ds-DNA, C3, C4, ANCA were within normal range. Osmotic fragility test was negative. Hb electrophoresis showed Hb-A1 of 91.8%, Hb-F of 5.7%, and Hb-A2 of 2.5%. Viral marker tests revealed negative cytomegalovirus (CMV) PCR and parvovirus antibody. The patient was referred to a hemato-oncologist with a diagnosis of AIHA and was subsequently treated with prednisolone. The patient’s parents did not mention any history of hospitalization or outpatient visits due to infectious disorders. Furthermore, according to her flow-cytometry results, low level of CD19+ and the very high level of CD56+ cells were detected. (Table 1). The immunophenotyping test were performed at the age of 2.5 years.

**Table 1:** The results of flow-cytometry and blood count

Laboratory Test	Values	Reference Values
CD3+ % (Absolute cell value)	40% (306)	39–73%
CD16+ % (Absolute cell value)	55% (420)	8.3–17.5%
CD45+ % (Absolute cell value)	88% (673)	
CD11b + (adhesion molecules)	Normal	
CD4+ % (Absolute cell value)	28% (214)	25–50%
CD8+ % (Absolute cell value)	8% (61)	11–32%
CD19+ % (Absolute cell value)	5% (38)	17–41%
CD14+ % (Absolute cell value)	12% (90)	3–6%
CD56+ (NK cells)	55	8.3–17.5
CD4/CD8	3.50	0.9–3.7
CD20+ % (Absolute cell value)	5% (42)	17–41%
Interferon γ receptor	Normal	
WBC count	3100	5000–15,500
Neutrophil % (Absolute cell value)	64.5% (2000)	
Lymphocyte % (Absolute cell value)	24.7% (765)	
Mix % (Absolute cell value)	10.8% (335)	
Hb (g/dL)	11.1	12–14
Plt (10^3^/mm^3^)	124	150–400
IgA (g/L)	1.234	0.13–1.02
IgG (g/L)	4.318	3.49–11.39
IgM (g/L)	0.967	0.40–2.29
DHR	180	> 50

As the patient was a result of a consanguineous marriage, a thorough family history was taken from her parents. Both her parents were in good health. The other sibling was a boy who presented with jaundice and anemia at the age of three months. He then presented with recurrent infections and passed away at the age of three years due to pneumonia. Serum PCR for CMV was positive in the deceased individual. No further clinical and laboratory data were available.

**Fig. 1:**
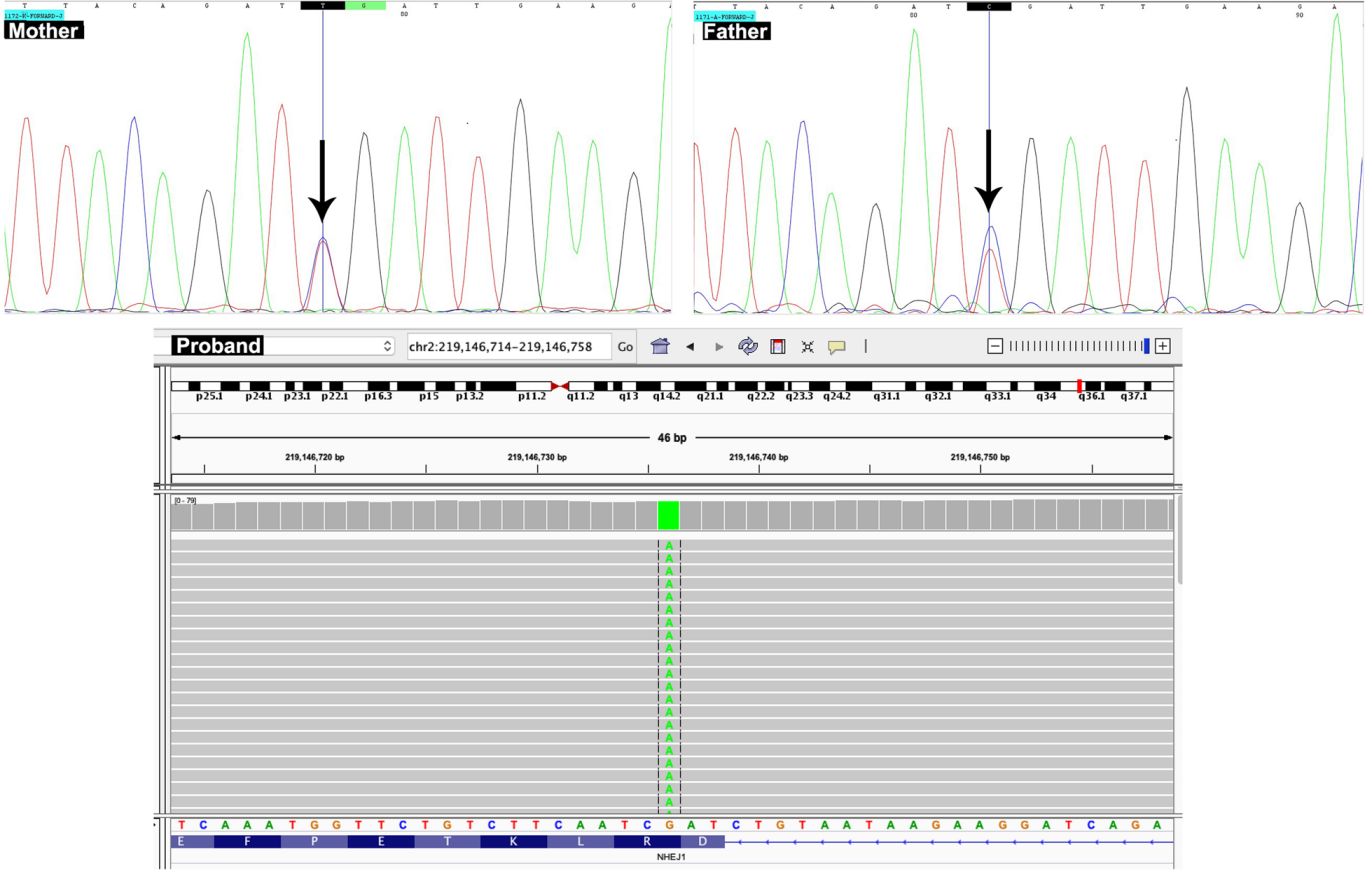
WES results showing homozygous mutation in *NHEJ1* gene in the proband, as visualized using Integrative Genome Viewer (IGV). Sanger sequencing confirms the presence of heterozygous mutation in *NHEJ1* gene in both parents. Arrows indicate the site of the mutation

To evaluate the patient for the underlying genetic disorder, whole-exome sequencing was carried out on the DNA extracted from the proband’s peripheral blood sample. Whole Exome Sequencing (WES) was performed on Illumina NextSeq500 instrument. The sequencing results were subsequently analyzed using different bioinformatics tools and databases such as BWA aligner, GATK and ANNOVAR. Whole exome sequencing details of coverage and number of reads are provided in Table 2. It was found that the patient had a stop-gain mutation in *NHEJ1* gene (NM_024782.2:c.532C > A). Sanger sequencing subsequently confirmed that the patient was homozygous and both parents were heterozygous for the mutation (Fig. 1).

**Table 2:** Whole Exome Sequencing detail of coverage and number of reads

Type	Value	Type	Value
Total Reads	74,338,832	Percent reads on target	46.45%
Passed filter Unique Reads aligned	74,253,527	Percent Passed filter Unique Reads aligned	99.89%
Mean Target Coverage	39.07	Percent on Target	46.45%
Percent Duplicate	22.38%	Percent duplicate in analysis	0%
Capture Method	Whole exome sequencing	Run method	NextSeq 500
GC content	44%	Sequence length	125
Nucleotide Covered GTE_1	98%	Nucleotide Covered GTE_5	87%
Nucleotide Covered GTE_8	79%	Nucleotide Covered GTE_10	75%
Nucleotide Covered GTE_15	65%	Nucleotide Covered GTE_20	58%
Nucleotide Covered GTE_30	47%	Nucleotide Covered GTE_40	38%
Nucleotide Covered GTE_50	31%	Nucleotide Covered GTE_60	24%
Nucleotide Covered GTE_70	19%	Nucleotide Covered GTE_80	14%
Nucleotide Covered GTE_90	10%	Nucleotide Covered GTE_100	8%

*GTE* Greater or equal to #

On follow-up, the patient had growth and development retardation with her length/height, head circumference and weight being below the 3rd percentile corrected for the age. Except for axillary lymphadenitis following BCG vaccination, the patient had had full vaccination including BCG, HepB, polio, MMR and DTP without any complications. On the last follow-up at the age of three years, the patient’s height and weight were 86 cm and 9.1 kg, respectively—both below the 3rd percentile corrected for the age. However, she has not had any evidence of immunodeficiency, despite living a normal life without any special precautions to preserve the patient’s health.

## Discussion and conclusion

Human cells are exposed to a wide variety of endogenous and exogenous DNA damaging agents. DSBs are considered one of the most severe forms of DNA damage, which could lead to cellular apoptosis or carcinogenesis, if left unrepaired [4, 6, 8]. DSBs are mostly repaired by either homologous recombination or non-homologous end-joining pathway [5]. Animal models evaluating the role of NHEJ pathway in the immune system have highlighted its importance in the immune system [11]. Animal models with defects in NHEJ have B and T lymphocyte maturation arrest and even embryonic lethality when accompanied by deficiencies in XRCC4/DNA-Ligase IV complex [12–14]. Given the vital role of repairing DSBs, mutation in any of the NHEJ genes, cause disruption in the immune system development, particularly B cell and T cell maturation, resulting in SCID [6, 8]. SCID presents early during the first few months of life and displays with severe bacterial and opportunistic infections, particularly respiratory infections [9].

In addition, it has been proposed that NHEJ1 has an important role in human cerebral cortex development [15]. Decreased expression of NHEJ1 has been shown to lead to defects in neuronal migration and decreased width of external cortical layers [4]. Furthermore, NHEJ deficiency appears to be a risk factor for the development of malignancy. Defects in NHEJ in P53-deficient mice have been shown to perpetually lead to development of pro-B cell lymphomas [16].

During the embryonic period, all cells are in a hyper-mitotic state. Therefore, mutations in *NHEJ1* commonly affect different cell types. Microcephaly, severe growth retardation, dysmorphic facial features and autoimmunity are reported alongside immunodeficiency [8, 17, 18]. To the best of our knowledge, the patient reported here is the first case with a homozygous pathogenic nonsense mutation (CADD score: 37) in *NHEJ1* gene with a competent immune system. This mutation would lead to the production of a protein lacking about one-third of its C-terminal amino-acid sequence.

We reported a patient with a pathogenic stop-gain mutation in *NHEJ1* who presented with AIHA, failure to thrive and microcephaly. However, she had no history of any bacterial or opportunistic infections. No previous history of respiratory infections, chronic diarrhea or any other complains was mentioned by her parents. The patient reported here had no history of prior hospital admission other than the one mentioned due to severe anemia. Buck, et al, reported a patient of Turkish origin with a similar mutation presenting with microcephaly and growth retardation and recurrent bacterial and opportunistic infections who had died at the age of four years due to septic shock [6]. Our findings highlight the importance of other end-joining auxiliary pathways such as polymerase θ-mediated end-joining, also known as a-EJ pathway [19, 20]. Although, due to the scarce number of individuals with deficiency in NHEJ, the exact role and function of a-EJ pathway is largely unknown, recent studies have emphasized its role in sustaining cell viability and genetic stability in case NHEJ is compromised.

All patients with defects in NHEJ reported to date, have been immunocompromised. Flow-cytometry in these patients demonstrates low T cell, very low or absent B cell and normal NK cell count. Serum immunoglobulin levels in these patients are generally low for IgG and IgA, and normal or high for IgM [3, 9, 18]. Our Patient had leukopenia with low level of CD19+ cells and very high level of CD56+ cells. In addition, IgA levels were mildly elevated. These findings might be a novel presentation. Further investigations to shed light on how these findings are compatible with immunocompetent phenotype are warranted. FTT and severe growth retardation have reported in all cases of NHEJ mutation [10]. Our patient’s weight, length/height and head circumference were below the 3rd percentile at birth and has been below the 3rd percentile on follow-up. Growth chart demonstrated that her growth pattern was not steady, as there were multiple periods of growth arrest. Her development was otherwise normal.

Microcephaly has been widely reported in previous studies, indicating the role of *NHEJ* gene in cerebral expansion [8]. *NHEJ* gene mutation leads to apoptotic death of post-mitotic neurons, causing CNS development issues, presenting itself with microcephaly, psychomotor retardation and ataxia [7, 17]. Our patient’s head circumference has always been below the 3rd percentile corrected for age. Her neurological development was otherwise normal and no finding in favor of developmental delay was noticed in her history or physical examination.

Live vaccines are absolutely contraindicated in patients with SCID as life-threatening complications could occur [21]. Our patient had complete vaccination record according to the national protocol including BCG, OPV and MMR without any complications except mild axillary lymphadenitis after BCG vaccination, which had resolved without medical intervention. Notably, vaccination against smallpox, chickenpox, influenza, and rotavirus is not part of Iran vaccination program.

Autoimmunity is common in previously reported patients with mutations in NHEJ genes; autoimmune cytopenia in particular is reported to be present in about a quarter of the patients [3, 22, 23]. The patient presented here was initially diagnosed with AIHA, but responded very well to oral prednisolone and required no further interventions.

In conclusion, our report highlights the importance of pathways other than NHEJ in DSB repair. Therefore, clinicians should be aware that pathogenic mutations in NHEJ pathway are not necessarily associated with clinical immunodeficiency.

## Abbreviations

ACLA: Anticardiolipin antibody
AIHA: Autoimmune hemolytic anemia
ANA: Antinuclear antibody
ANCA: Anti-neutrophil cytoplasmic antibody
BCG: Bacillus Calmette–Guérin
CMV: Cytomegalovirus
DSB: Double-strand break
Ds-DNA: Double stranded DNA
DTP: Diphtheria, tetanus and pertussis
FTT: Failure to thrive
MMR: Measles, mumps, and rubella
NHEJ: Non-homologous end-joining
SCID: Severe combined immunodeficiency
WBC: White blood cell

## References

[1] Lieber MR (2010). The mechanism of double-strand DNA break repair by the nonhomologous DNA end-joining pathway. Annu Rev Biochem.

[2] Lieber MR, Karanjawala ZE (2004). Ageing, repetitive genomes and DNA damage. Nat Rev Mol Cell Biol.

[3] Gellert M (1994). DNA double-strand breaks and hairpins in V(D)J recombination. Semin Immunol.

[4] El Waly B, Buhler E, Haddad MR, Villard L (2015). Nhej1 deficiency causes abnormal development of the cerebral cortex. Mol Neurobiol.

[5] Sancar A, Lindsey-Boltz LA, Unsal-Kacmaz K, Linn S (2004). Molecular mechanisms of mammalian DNA repair and the DNA damage checkpoints. Annu Rev Biochem.

[6] Buck D, Malivert L, de Chasseval R, Barraud A, Fondaneche MC, Sanal O, Plebani A, Stephan JL, Hufnagel M, le Deist F (2006). Cernunnos, a novel nonhomologous end-joining factor, is mutated in human immunodeficiency with microcephaly. Cell.

[7] Revy P, Malivert L, de Villartay JP (2006). Cernunnos-XLF, a recently identified non-homologous end-joining factor required for the development of the immune system. Curr Opin Allergy Clin Immunol.

[8] Cagdas D, Ozgur TT, Asal GT, Revy P, De Villartay JP, van der Burg M, Sanal O, Tezcan I (2012). Two SCID cases with Cernunnos-XLF deficiency successfully treated by hematopoietic stem cell transplantation. Pediatr Transplant.

[9] Sheikh F, Hawwari A, Alhissi S, Al Gazlan S, Al Dhekri H, Rehan Khaliq AM, Borrero E, El-Baik L, Arnaout R, Al-Mousa H (2017). Loss of NHEJ1 protein due to a novel splice site mutation in a family presenting with combined immunodeficiency, microcephaly, and growth retardation and literature review. J Clin Immunol.

[10] Turul T, Tezcan I, Sanal O (2011). Cernunnos deficiency: a case report. J Investig Allergol Clin Immunol.

[11] de Villartay JP, Fischer A, Durandy A (2003). The mechanisms of immune diversification and their disorders. Nat Rev Immunol.

[12] Bryans M, Valenzano MC, Stamato TD (1999). Absence of DNA ligase IV protein in XR-1 cells: evidence for stabilization by XRCC4. Mutat Res.

[13] Dudley DD, Chaudhuri J, Bassing CH, Alt FW (2005). Mechanism and control of V(D)J recombination versus class switch recombination: similarities and differences. Adv Immunol.

[14] Revy P, Buck D, le Deist F, de Villartay JP (2005). The repair of DNA damages/modifications during the maturation of the immune system: lessons from human primary immunodeficiency disorders and animal models. Adv Immunol.

[15] Cantagrel V, Lossi AM, Lisgo S, Missirian C, Borges A, Philip N, Fernandez C, Cardoso C, Figarella-Branger D, Moncla A (2007). Truncation of NHEJ1 in a patient with polymicrogyria. Hum Mutat.

[16] Ferguson DO, Alt FW (2001). DNA double strand break repair and chromosomal translocation: lessons from animal models. Oncogene.

[17] Berthet F, Caduff R, Schaad UB, Roten H, Tuchschmid P, Boltshauser E, Seger RA (1994). A syndrome of primary combined immunodeficiency with microcephaly, cerebellar hypoplasia, growth failure and progressive pancytopenia. Eur J Pediatr.

[18] Cipe FE, Aydogmus C, Babayigit Hocaoglu A, Kilic M, Kaya GD, Yilmaz Gulec E: Cernunnos/XLF deficiency: a syndromic primary immunodeficiency. Case Rep Pediatr 2014, 2014:614238. 10.1155/2014/614238 PMC3910469 24511403

[19] Wyatt DW, Feng W, Conlin MP, Yousefzadeh MJ, Roberts SA, Mieczkowski P, Wood RD, Gupta GP, Ramsden DA (2016). Essential roles for polymerase theta-mediated end joining in the repair of chromosome breaks. Mol Cell.

[20] Chang HHY, Pannunzio NR, Adachi N, Lieber MR (2017). Non-homologous DNA end joining and alternative pathways to double-strand break repair. Nat Rev Mol Cell Biol.

[21] Marciano BE, Huang CY, Joshi G, Rezaei N, Carvalho BC, Allwood Z, Ikinciogullari A, Reda SM, Gennery A, Thon V (2014). BCG vaccination in patients with severe combined immunodeficiency: complications, risks, and vaccination policies. J Allergy Clin Immunol.

[22] Notarangelo LD. Primary immunodeficiencies (PIDs) presenting with cytopenias. Hematology Am Soc Hematol Educ Program. 2009:139–43. 10.1182/asheducation-2009.1.139 20008192

[23] S A, M N, Bemanian MH, Shakeri R, Taghvaei B, Ghalebaghi B, Babaie D, Bahrami A, Fallahpour M, Esmaeilzadeh H (2016). Phenotyping and follow up of forty-seven Iranian patients with common variable immunodeficiency. Allergol Immunopathol (Madr).

